# Gut-spine axis: a possible correlation between gut microbiota and spinal degenerative diseases

**DOI:** 10.3389/fmicb.2023.1290858

**Published:** 2023-10-27

**Authors:** Tadatsugu Morimoto, Takaomi Kobayashi, Toshihiko Kakiuchi, Motohiro Esaki, Masatsugu Tsukamoto, Tomohito Yoshihara, Hirohito Hirata, Shoji Yabuki, Masaaki Mawatari

**Affiliations:** ^1^Department of Orthopedic Surgery, Faculty of Medicine, Saga University, Saga, Japan; ^2^Department of Pediatrics, Faculty of Medicine, Saga University, Saga, Japan; ^3^Division of Gastroenterology, Department of Internal Medicine, Faculty of Medicine, Saga University, Saga, Japan; ^4^Fukushima Medical University School of Health Sciences, Fukushima, Japan

**Keywords:** gut microbiota, gut dysbiosis, spinal degenerative disease, inflammation, gut-spine axis, gut-bone axis

## Abstract

As society ages, the number of patients with spinal degenerative diseases (SDD) is increasing, posing a major socioeconomic problem for patients and their families. SDD refers to a generic term for degenerative diseases of spinal structures, including osteoporosis (bone), facet osteoarthritis (joint), intervertebral disk degeneration (disk), lumbar spinal canal stenosis (yellow ligament), and spinal sarcopenia (muscle). We propose the term “gut-spine axis” for the first time, given the influence of gut microbiota (GM) on the metabolic, immune, and endocrine environment in hosts through various potential mechanisms. A close cross-talk is noted between the aforementioned spinal components and degenerative diseases. This review outlines the nature and role of GM, highlighting GM abnormalities associated with the degeneration of spinal components. It also summarizes the evidence linking GM to various SDD. The gut-spine axis perspective can provide novel insights into the pathogenesis and treatment of SDD.

## 1. Introduction

With an increasing aging population in society, the number of patients with spinal degenerative diseases (SDD) has been on the rise, with a reported annual prevalence of 27.1% for SDD in the United States (Parenteau et al., 2021). SDD refers to a generic term for diseases caused by the degeneration of spinal structures. Examples of combinations of spinal structures and their degenerative diseases include osteoporotic vertebral fractures (OVF) in bones, facet joint osteoarthritis (OA) in cartilages, intervertebral disk degeneration (IVDD) in the intervertebral disk (IVD), lumbar spinal canal stenosis (LSS) in ligaments, and spinal sarcopenia in muscles. Furthermore, osteoporosis, facet joint OA, IVDD, LSS, and spinal sarcopenia tend to coexist, as their incidence increases with age (Parenteau et al., 2021), adversely affecting each other’s pathology and forming a negative spiral cycle.

Several studies have reported risk factors, including aging, heavy labor (mechanical stress), trauma, genetics, obesity, and metabolic syndrome, for SDD (Puenpatom and Victor, 2009; Yoshimura et al., 2012; Imajo et al., 2015; Chen et al., 2019; Guss et al., 2019; Azzini et al., 2020; Kuo et al., 2020; Parenteau et al., 2021; Li et al., 2022). The pain and paralysis caused by SDD have become a major problem that affects the patient’s life and work and imposes a significant economic burden on the patient, family, and society (Imajo et al., 2015). The causes of SDD remain poorly understood, and understanding their causes can be critical for prevention and treatment. The progressive degeneration of spinal structures has been attributed not only to biomechanical injury or stress but also to biochemical stressors that can adversely affect the regular activity of cells and tissues in spinal structures. These risk factors, independently or complexly linked, contribute to the complex interplay between mechanical and biochemical factors that lead to the etiology of SDD.

Metabolic syndrome involves multiple physiological systems that are directly related to the presence of four major clusters: insulin resistance, obesity, vascular pathology, and dyslipidemia (Azzini et al., 2020). Metabolic syndrome induces “meta-inflammation,” characterized by persistent, low-grade systemic inflammation caused by metabolic stress, which places biochemical stress on systemic tissues (Azzini et al., 2020). In particular, in metabolic syndrome, chronic low-level inflammation mediated by macrophages occurs in the liver, visceral fat, pancreas, colon, brain, and musculoskeletal tissues and is implicated in the development of various diseases (Azzini et al., 2020). In addition, chronic low-level persistent tissue inflammation that occurs with aging, also referred to as “inflammaging,” has been reported to be associated with the development of osteoporosis (Benoist, 2003; Ibáñez et al., 2019; Zaiss et al., 2019), IVDD (Benoist, 2003; Franceschi and Campisi, 2014; Sadowska et al., 2018; Li et al., 2022), OA (Berthelot et al., 2019; De Sire et al., 2020; Favazzo et al., 2020; Gracey et al., 2020; Qaiyum et al., 2021; Zaiss et al., 2021; Motta et al., 2023; Romero-Figueroa et al., 2023), and sarcopenia (Picca et al., 2018; Ticinesi et al., 2019; Kuo et al., 2020; Przewłócka et al., 2020). The gut microbiota (GM), a complex of intestinal bacterial populations, is responsible for a series of metabolic, immune, structural, and neurological functions, including metabolic homeostasis, immune system development and maturation, resistance to infection, and neurotransmitter production (Biver et al., 2019). Recent studies have highlighted important regulatory functions of GM in neuroendocrine and immune functions through the activity of microbiome and its metabolites and its involvement in disease processes in various organs inside and outside the gut (such as brain, kidney, liver, heart, musculoskeletal) (Biver et al., 2019). GM is also involved in the development and progression of inflammatory diseases such as obesity and metabolic syndrome. GM disruption has emerged as a hidden risk factor inducing the production of inflammatory cytokines and bacterial metabolites, which may be involved in the pathophysiological mechanisms of musculoskeletal diseases, including SDD (Biver et al., 2019).

Although the gut-brain axis is the best-known term for this cross-talk between GM and the gut and distant organs, data supporting the important role of GM in spinal conditions and its involvement in the onset and progression of SDD have been obtained. A gut-bone axis (Ibáñez et al., 2019; Zaiss et al., 2019), gut-joint axis (Berthelot et al., 2019; De Sire et al., 2020; Favazzo et al., 2020; Gracey et al., 2020; Qaiyum et al., 2021; Zaiss et al., 2021; Romero-Figueroa et al., 2023), gut-disk axis (Li et al., 2022), and gut-muscle axis (Picca et al., 2018; Ticinesi et al., 2019; Kuo et al., 2020; Przewłócka et al., 2020) have also been proposed, with possible association between GM and SDD-related pain (Tonelli Enrico et al., 2022). Furthermore, studies have also reported a relationship between GM and adolescent idiopathic scoliosis (Shen et al., 2019) and ankylosing spondylitis (Guggino et al., 2021). Given the close relationship between GM and SDD based on the common factors of immunity, metabolism, and inflammation, we can hypothesize the involvement of GM in the degeneration of spinal structures, with GM being a potential mechanism for the development of SDD.

We propose the novel and comprehensive concept of the “gut-spine axis” for the first time, as knowledge about the “gut-spine cross-talk,” which summarizes the relationship of GM with spinal structures (bone, cartilage, disks, ligaments, and muscle) and the impact of GM on pain can improve our understanding of the etiology of SDD and contribute to development of treatment strategies for SDD. This review outlines the nature and role of GM and GM abnormalities associated with the degeneration of spinal structures and also summarizes the evidence linking GM to various SDD. For a broader, more flexible, and more comprehensive organization, we employed a narrative review approach and analyzed several important articles regarding the relationship between SDD and GM published in peer-reviewed scientific journals. This study outlines the reports supporting the presence of a gut-spine axis in the etiology of SDD.

## 2. Gut microbiota and host interaction

### 2.1. Characteristics of GM

Surprisingly, the large intestine contains more than 70% of all the microorganisms in the human body (Jandhyala et al., 2015). GM refers to the diverse collection of microorganisms, including commensal, symbiotic, and pathogenic species, that reside in the intestine (Jandhyala et al., 2015). The intestine, being a multicellular organ acquired at birth, exists as a distinct entity yet has clearly co-evolved with the human genome. It interacts with the host and exerts various influences on it through communication and other mechanisms (Qin et al., 2010). Although there are more than 1,000 species of bacteria and 10^14^ bacterial cells in the human gut (Chen et al., 2022b), detection of most anaerobic bacteria has been difficult using culture techniques. In recent years, a new sequencing technology, known as high-throughput “next-generation sequencing” (NGS) of microbial DNA, has emerged as a leading approach to better characterize the human microbiota (Chen et al., 2022b). GM samples for analysis are collected from the stool. Microbiome profiling is often performed by sequencing 16S rRNA gene amplicons in a culture-independent method or by shotgun sequencing (metagenomics) of the entire microbiome. As a result, GM has gained significant attention due to advancements in NGS technology. The importance of GM’s diversity, composition, and functional characteristics in shaping human health has been widely acknowledged.

Under normal physiologic conditions, GM plays a fundamental role in various aspects of host physiology. This includes functions such as nutrition and metabolism, maintaining the integrity of the gut barrier to prevent pathogen invasion, and supporting immune responses. In contrast, disruption of these roles may contribute to inflammation, pain, and development of various diseases (Jandhyala et al., 2015).

### 2.2. Role of GM in nutrition, metabolism, and immunomodulation related to SDD

**Figure 1:**
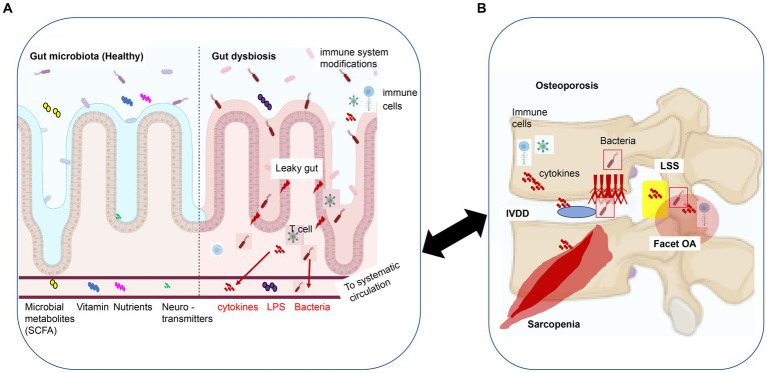
The gut-spine axis: a model of intestinal epithelial damage signaling that may regulate degeneration of spinal structures. **(A)** Gut microbiota and dysbiosis: GM supplies the host with essential functions such as the synthesis of short-chain fatty acids (SCFA), vitamins, nutrients, and neurotransmitters (including some precursor substances). When leaky gut syndrome occurs, which results in altered GM composition and GM dysbiosis, the intestinal barrier is compromised, altering physiological and metabolic functions and disrupting immune, endocrine, vascular, and nervous system responses, resulting in bacteremia, bacterial-derived compounds (LPS), and cytokines circulating along the bloodstream to systemic tissues. **(B)** Spinal degenerative diseases (SDD): osteoporosis, IVDD, facet OA, and LSS. GM dysbiosis-derived metabolites, bacteremia, and inflammatory cytokines can result in local and systemic responses that may cause SDD. This figure was adapted and modified from the figure by Li et al. (2022) and was created using BioRender.com. This content is available under Creative Commons Attribution 4.0 International License (https://link.springer.com/article/10.1007/s00586-022-07152-8). IVDD, intervertebral disk degenerative disease; OA, osteoarthritis; LSS, lumbar spinal canal stenosis; SCFA, short-chain fatty acids; LPS, lipopolysaccharide.

GM provides the host with vital functions, including the fermentation of dietary fiber and indigestible polysaccharides, leading to the production of biotransformed bile acids that regulate calcium absorption. Additionally, GM is involved in the synthesis of vitamins B and K, as well as the production of essential and nonessential amino acids, short-chain fatty acids (SCFA) (i.e., acetic acid, propionic acid, and butyric acid), and neurotransmitters (including some precursors) (Ibrahim et al., 2022, p. 191; Chen et al., 2022b; Figure 1A). GM is often referred to as an endocrine organ capable of influencing the function of distant organs and systems, given the involvement of GM in the production of metabolites (Clarke G. et al., 2014).

Naturally, the absorption of calcium and the production of vitamin K and amino acids, both of which involve GM, are essential for musculoskeletal health (Chen et al., 2022b). In addition, because GM secretes estrogen-regulating enzymes, aberrations in GM, such as low diversity of GM, may lead to a decrease in circulating estrogen level (Baker et al., 2017). Estrogen deficiency at menopause is a major cause of postmenopausal osteoporosis, with excessive osteoclast formation and bone resorption stimulation due to estrogen deficiency at menopause leading to rapid bone loss (D’Amelio et al., 2008). Decreased estrogen levels increase the production of inflammatory and osteoclastogenic cytokines, leading to osteoporosis. There is speculation that postmenopausal women, who lose the immunosuppressive effects of estrogen, may experience greater bone loss due to an increased inflammatory state caused by an unfavorable GM composition (Baker et al., 2017). Thus, the interaction between GM and estrogen influences the composition of GM and the response of host tissues, including bone, to GM (Baker et al., 2017). Thus, the interaction between GM and estrogen is also referred to as the “estrogen-gut microbiome axis” (Baker et al., 2017).

The analgesic, anti-inflammatory, antinociceptive, and neuroprotective effects of the B vitamins have been widely documented. Among the B vitamins, vitamin B12 is known to be effective in reducing back pain and nerve pain (Tonelli Enrico et al., 2022).

SCFA are largely secreted in the colon by GM through anaerobic fermentation of dietary fiber and have a critical role in regulating intestinal inflammation and epithelial barrier function. In addition, SCFA enters the bloodstream and further contributes to the regulation of systemic inflammation (Tonelli Enrico et al., 2022). Besides the immune system controlling inflammation, SCFA influences various aspects of metabolism, including bone metabolism, glucose metabolism, brown adipose tissue activation, liver mitochondrial function regulation, whole body energy homeostasis, appetite, and sleep regulation (Lucas et al., 2018). Neurotransmitters such as dopamine, serotonin, leptin, noradrenaline, glutamate, and GABA can be directly produced by GM or indirectly regulated by GM (Tonelli Enrico et al., 2022).

At least 50% of dopamine is produced in the gut and has regulatory functions in systemic inflammation and chronic pain through its involvement in immunity responses (Vidal and Pacheco, 2020). A previous study has reported that 90% of serotonin is produced in the intestinal tract and that circulating serotonin of intestinal origin inhibits bone formation and decreases bone mass (Yadav et al., 2008). The GM was shown to regulate intestinal chromaffin cells that modulate serotonin production in the gut (Yadav et al., 2008). Serotonin also regulates physiological functions related to pain (Malinova et al., 2018). Leptin, an adipocyte-specific hormone, regulates bone formation, while GM positively regulates systemic levels of leptin and is involved in bone metabolism (Charnay et al., 2000).

Noradrenaline is also responsible for pain and inflammation (Tonelli Enrico et al., 2022). Glutamate and GABA function as major excitatory and inhibitory neurometabolites, respectively, in the central nervous system and play various important physiological roles, including processing and regulation of pain (e.g., inflammation; Tonelli Enrico et al., 2022).

Consecutively, vitamin B, SCFA, and neurotransmitters are involved in the immune response and regulate pain and inflammation. Therefore, GM-related changes in nutrition and metabolites can impact musculoskeletal health, reduce anti-inflammatory effects globally, and lower pain tolerance through the gut-brain axis. These factors may contribute to the development of SDD and SDD-related pain.

Conversely, lipopolysaccharide (LPS) from gram-negative bacteria, a metabolite derived from GM, is an inflammatory metabolite that acts on macrophages, which maintain tissue homeostasis and exert pro-inflammatory effects (Burcelin et al., 2013). LPS enters the systemic circulation through the intestinal tract and bloodstream to induce low-level systemic inflammation. LPS levels are known to be elevated in chronic inflammatory diseases such as obesity (Burcelin et al., 2013), metabolic syndromes (Burcelin et al., 2013), osteoporosis (Wu et al., 2021), OA (Huang and Kraus, 2016), and IVDD (Lisiewski et al., 2022).

Therefore, while some molecules produced by intestinal bacteria are beneficial, others are harmful and can affect endocrine cells in the gut, the enteric nervous system, intestinal permeability, and the immune system (Ohlsson and Sjögren, 2015). Meanwhile, GM composition and function may have beneficial or detrimental effects on the degeneration of spinal structures (such as bones, cartilage, disks, ligaments, and muscles) and pain associated with SDD (Charles et al., 2015; Chen et al., 2022b; Figure 1B).

### 2.3. Dysbiosis of GM

#### 2.3.1. Change in GM composition

**Figure 2:**
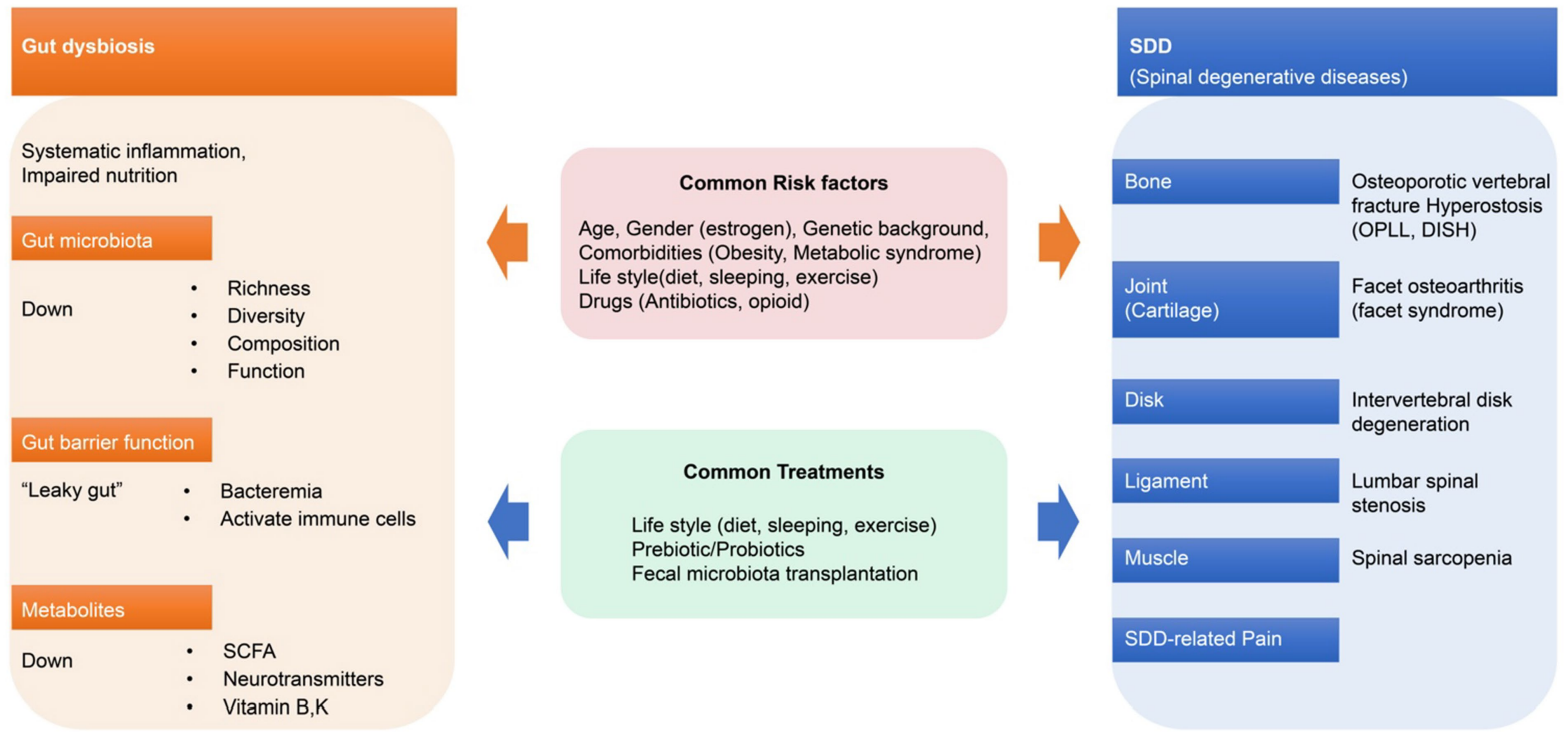
Gut dysbiosis and spinal degenerative diseases. Gut dysbiosis and SDD have common risk factors and treatments that can interact with each other. OPLL, ossification of the posterior longitudinal ligament; DISH, diffuse idiopathic skeletal hyperostosis.

GM is acquired at birth, derived almost exclusively from the mother (Das and Nair, 2019; Chen et al., 2022a). In the case of healthy people, the characteristic features of GM are a state of dynamic homeostasis defined by the richness and diversity of GM compositions and by its stability and resilience to withstand various types of disturbances (Das and Nair, 2019). The richness and diversity of GM compositions can be altered by environmental factors (such as aging) and lifestyle factors (such as diet, sleep, and exercise; Ohlsson and Sjögren, 2015; Fei et al., 2021; Figure 2).

#### 2.3.2. Effects of aging on GM composition

Since GM composition continues to change in an age-dependent pattern from infancy to old age as GM co-evolves with the host (Chen et al., 2022a), organismal aging is inevitably accompanied by changes in GM. GM in older adults is characterized by reduced diversity and stability, decreased expression of genes producing SCFA, reduced ability to degrade glycoconjugates, enhanced proteolytic function, and at the genus level, enrichment of Proteobacteria (including many members of opportunistic pathogenic bacteria), and an increased proportion of the *Bacteroidetes* and *Clostridium* genera (Buford, 2017). Notably, reduced diversity of GM may correlate with “frailty index,” an indicator of biological age and a predictor of healthy life expectancy (Ke et al., 2021).

Furthermore, aging not only affects the composition of GM but also is associated with changes in the gut lumen and barrier function, including shrinkage of the protective mucus layer of the gut, loss of gut tight junction proteins, and increased permeability of the epithelial barrier (Elderman et al., 2017). Several lines of evidence in this regard suggest that low-level systemic inflammation has recently been recognized as a phenomenon associated with older adults (Konturek et al., 2015; Borghesan et al., 2020). Meanwhile, in healthy centenarian GM, the anti-inflammatory effect of *Faecalibacterium* spp. is strong, and blood LPS level is low, indicating that *Faecalibacterium* spp. may be suppressing host inflammation (Park et al., 2015).

In recent years, a cellular senescence-centric view of the aging process has emerged. Cellular senescence results in senescent cells (SCs), which exhibit age-dependent accumulation in tissues and organs of various mammalian species, including rodents and primates (Sharma, 2022). The surviving senescent cells develop a senescence-associated secretory phenotype (SASP), which secretes various inflammatory cytokines and promotes chronic inflammation and carcinogenesis (Tuttle et al., 2021). Low levels of chronic inflammation occur in older adults, which has been partly attributed to cellular senescence (Herbig et al., 2006). Increased SC burden with aging destroys tissue structure and function and is emerging as an important factor in increasing disease risk and mortality in older adults (Burd et al., 2013). Osteoporosis, sarcopenia, IVDD, and OA have been reported as musculoskeletal diseases associated with SASP (Burd et al., 2013; Khosla et al., 2020; Tuttle et al., 2021).

Increased age-related oxidative/inflammatory stress contributes to SC accumulation, and the application of antioxidants has been shown to inhibit cellular senescence both *in vitro* and *in vivo* (Kumar et al., 2019; Varela-Eirín et al., 2020). Therefore, it is conceivable that neutralizing oxidative/inflammatory stressors may attenuate or delay SC development and accumulation. In a landmark study, removing SC load in aging tissues delayed the onset and severity of age-related conditions (Baker et al., 2011). Since then, several natural and synthetic compounds that selectively target SC, called “senolytics,” have been identified. Clinical trials based on senolytics have already shown promising results in countering the harmful effects of aging (Baker et al., 2011; Kumar et al., 2019; Varela-Eirín et al., 2020).

Various metabolites of GM are known to exert strong anti-inflammatory and antioxidant properties and may be useful in the prevention of inflammatory and tumorigenic environments associated with aging (Chang et al., 2014; De Marco et al., 2018; Wang et al., 2018; Riaz Rajoka et al., 2019; Chung et al., 2020; Rossi et al., 2020; Sharma, 2022). In contrast, GM dysbiosis can also cause chronic inflammatory stress throughout the body, which may promote cellular senescence. GM dysbiosis may also affect SDD via cellular senescence. Thus, GM is closely related to human aging, and changes in GM may predict the development and prognosis of SDD.

#### 2.3.3. Effects of lifestyle (i.e., diet, sleep, exercise) on GM composition

Diet, sleep, and exercise are three key components of a healthy lifestyle that can affect GM composition and function (Ohlsson and Sjögren, 2015; Fei et al., 2021; Figure 2). Diets can significantly change GM composition depending on nutrients. This can be attributed to the fact that nutrients can alter the GM microenvironment, including GM composition, metabolism, and the host’s immune response (Li et al., 2016).

Sleep length has been demonstrated to have a significant effect on the composition and function of GM (Fei et al., 2021). Short sleep duration promotes GM abnormalities (less diversity of gut bacteria and fewer anti-inflammatory gut bacteria), which may affect chronic inflammation-related diseases (Benedict et al., 2016; Poroyko et al., 2016; Reynolds et al., 2017; Fei et al., 2021).

Sleep disruption has been reported to affect physical, mental, and emotional functioning, in addition to hormonal and metabolic disturbances (Fei et al., 2021). Possible causes include increased inflammatory markers (Leproult et al., 2014), increased sympathetic nervous system activity, abnormal cortisol rhythms, and changes in appetite-regulating hormones and food intake (Spiegel et al., 1999), all of which contribute to the risk of diabetes and obesity. Given the association between diseases associated with sleep disorders and diseases derived from abnormalities in the GM, the adverse health effects observed in sleep disorders may be partly due to gut bacteria (Fei et al., 2021).

Physical activity (including exercise) has been shown to produce positive changes in the qualitative and quantitative composition and metabolic function of GM and provide health benefits to the host in animal models (Li et al., 2016; Poroyko et al., 2016; Reynolds et al., 2017) and humans (Clarke S. F. et al., 2014). Athletes generally have a high diversity of GMs with anti-inflammatory properties and a high capacity for SCFA synthesis compared to sedentary controls (Barton et al., 2018; Ticinesi et al., 2019). In studies including both younger and older adults, the expression of *Bifidobacterium* spp. and *Faecalibacterium prausnitzii* involved in SCFA production, which potentiates anti-inflammatory effects, increased after exercise implementation (aerobic and resistance training), and the concentration of butyrate in the stool increased (Allen et al., 2018; Erlandson et al., 2021). Regular exercises have been shown to benefit older adults, especially those who are overweight, by maintaining the stability (composition and function) of the intestinal microbiota (Zhu et al., 2020). The utility of exercise in preventing and treating SDD is not limited to the typical rehabilitative effects, such as improved muscle function, pain relief, and improved range of motion, but also to the synergistic effects of improving GM composition and function with improved health benefits for the host.

#### 2.3.4. Causes and effects of GM dysbiosis

Aging, dietary changes, smoking and alcohol consumption, disease, antibiotic treatment, and pathogens can alter GM composition, leading to dysbiosis, defined as detrimental changes in bacterial composition, diversity, and function (Zheng et al., 2020). Dysbiosis can lead to imbalances in metabolic and immune regulatory networks that usually suppress intestinal inflammation, leading to inflammation, tissue destruction, and disease development (Kinashi and Hase, 2021).

The so-called “leaky gut syndrome,” which is characterized by altered permeability of the intestinal wall to antigens, is an example of dysbiosis, leading to systemic inflammation and abnormal immune response (Kinashi and Hase, 2021). When leaky gut syndrome, an example of dysbiosis, occurs, GM loses its protective capacity, the intestinal barrier is compromised, diffusion of bacterial-derived compounds is enhanced, and metabolite and cytokine expression is altered (Ohlsson and Sjögren, 2015; Kinashi and Hase, 2021). Therefore, it is not impossible for dysregulated circulatory inflammatory cytokines to reach spinal structures (bones, cartilage, disks, ligaments, and muscles; Figures 1A,B, 2) and disrupt normal cell signaling and metabolic activity.

In ophthalmology, GM dysbiosis reportedly causes chronic low-grade inflammation, which is characteristic of inflammatory conditions with increased intestinal permeability and increased production of IL-6, IL-1β, TNF-α, and VEGF-A, ultimately leading to exacerbation of pathological angiogenesis in choroidal neovascularization (Andriessen et al., 2016). Similar mechanisms may exist in the pathological angiogenesis of various diseases, including SDD. Dysbiosis, with an altered composition of GM, can alter gut wall permeability and physiological and metabolic functions, and disrupt immune, endocrine, vascular, and nervous system responses, ultimately leading to: diseases characterized by immune dysregulation (allergies, autoimmune diseases, inflammatory; Miyauchi et al., 2023), metabolic diseases (diabetes, metabolic syndrome; Guss et al., 2019), cardiovascular diseases (coronary artery disease; Jie et al., 2017), neurodegenerative diseases (autism; Cryan et al., 2019), cancer (stomach, lung, colon; DeStefano Shields et al., 2021; Sepich-Poore et al., 2021), and even SDD such as osteoporosis, facet joint OA, IVDD, and LSS (Ohlsson and Sjögren, 2015; Azzini et al., 2020; DeStefano Shields et al., 2021; Kinashi and Hase, 2021; Li et al., 2022; Lisiewski et al., 2022; Figure 2).

Furthermore, the reduced number of myeloid progenitor cells in sterile mice suggests that the metabolites of intestinal bacteria affect immune cells in the bone marrow (Khosravi et al., 2014). The human spine has 26 vertebrae that are the source of immune cells. GM dysbiosis can cause abnormal immune cell formation in the spinal bone marrow, leading to systemic inflammation and back pain (Nouh and Eid, 2015). To summarize, the role and relevance of GM in the pathogenesis of a group of age-related chronic inflammation-related diseases is an important emerging phenomenon that should not be overlooked.

#### 2.3.5. Are the spinal structures (disks, joints) sterile?

Culture-independent approaches such as 16S rRNA sequencing and shotgun metagenomics have expanded our ability to identify all bacteria, confirming the presence of bacteria at sites previously considered sterile (Ozkan and Willcox, 2019). As a result, microbiomes are present in sites outside of joint environments traditionally thought to be sterile, including the reproductive tract, sperm, fetus, breast, and eye (Ardissone et al., 2014; Ozkan and Willcox, 2019).

During the neovascularization phase in OA, there is evidence that bacteria and bacterial products have a greater tendency to enter cartilage and subchondral bone from the blood; this is particularly notable as these areas are typically less vascularized (Pesesse et al., 2011). Certainly, the microbiome is present in knee and hip OA (Dunn et al., 2020). In addition, a systematic review reported the presence of various bacteria in degenerated and Modic change-altered IVD (Granville Smith et al., 2022).

In dysbiosis, due to the impaired barrier function of the intestinal wall and immune system, some GM may migrate from the intestinal tract to the bloodstream and cause transient bacteremia (Fine et al., 2020), allowing some GM to settle directly in the joints or allowing leukocytes and macrophages (Miller et al., 2020) to reach the joints and IVD as “Trojan horses” for the bacteria (Alverdy et al., 2020). This may result in the migration of some GM in the subchondral bone marrow and deep in the cartilage or disks, which may directly contribute to the deformity changes (Chisari et al., 2021; Rajasekaran et al., 2022). Chronic inflammation and adverse immune effects due to GM dysbiosis and bacteremia may also contribute to postoperative infection for SDD.

## 3. Gut-spine axis

GM influences host metabolism, immunity, the endocrine environment, and the gut-brain-bone axis. It also affects spinal structures (bone, cartilage, disks, ligaments, and muscles) through a variety of potential mechanisms (Behera et al., 2020; Seely et al., 2021; Figures 1A,B).

GM dysbiosis affects the health of spinal structures through the following three mechanisms: (1) nutrition, including calcium, amino acids, and vitamin K; (2) immune regulation related to estrogen, SCFA, and systemic inflammation; and (3) neurotransmitters such as serotonin and leptin that have been demonstrated to affect bone metabolism, ultimately causing an imbalance between osteoblasts and osteoclasts (Chen et al., 2022b). Additionally, increased inflammatory stress associated with GM dysbiosis promotes the senescence of spinal musculoskeletal cells and the accumulation of various SCs at spinal structures (Borghesan et al., 2020; Sharma, 2022). SCs may cause local and systemic inflammation *via* SASP and contribute to the development of the following SDD: osteoporosis for senescent osteocytes and osteoblasts, OA for senescent chondrocytes, and IVDD for senescent nucleus pulposus cells (Borghesan et al., 2020; Sharma, 2022). This chapter outlines the nature and role of GM and dysbiosis associated with the degeneration of spinal structures (bone, cartilage, ligaments, disks, and muscles) and contributing to the development and progression of SDD and SDD-derived pain.

### 3.1. Gut-bone axis: osteoporosis and hyperostotic diseases

**Figure 3:**
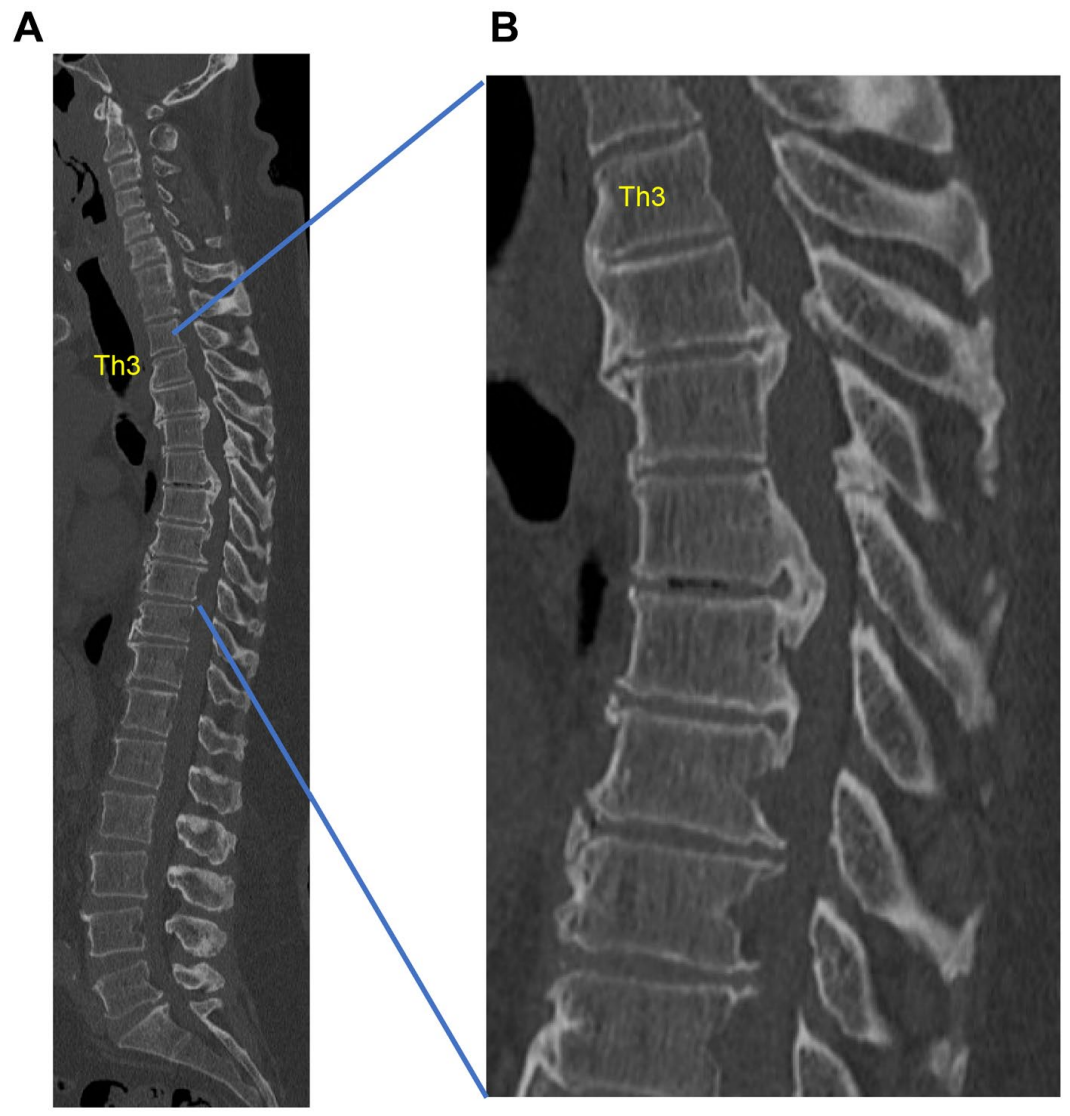
Example of thoracic ossification of the posterior longitudinal ligament (OPLL) in a 53-year-old obese male with type 2 diabetes mellitus. The spinal canal is markedly narrowed by thoracic OPLL, with inability to walk. **(A)** Sagittal computed tomography (CT) of the whole spine. **(B)** Sagittal CT of the thoracic spine. This is a case from our institution.

SDD related to bone metabolism includes OVF and hyperostotic diseases [i.e., ossification of posterior longitudinal ligament (OPLL) and diffuse idiopathic skeletal hyperostosis (DISH)] (Figure 3). Osteoporosis and hyperostotic diseases have an immune-inflammatory mechanism involved in their pathogenesis (Zaiss et al., 2019; Mader et al., 2021; Kawaguchi, 2022). Bone is an organ that depends on the dynamic balance between osteoblasts and osteoclasts to maintain normal function.

Inflammatory diseases involve several inflammatory cytokines that play a role in regulating osteoblasts and osteoclasts, and immune activity is considered a significant risk factor for osteoporosis (Arron and Choi, 2000). The term “osteoimmunology” describes the close interaction between the immune system and bone metabolism and the role of immune cells or immune-related factors in the regulation of skeletal development (Takayanagi, 2021).

In recent years, the effects of GM on bone tissue have been confirmed in animals lacking GM (Axenic mice), in animals fed GM-modifying antibiotics and diet, and in humans (Ibáñez et al., 2019). In addition, the presence of osteoporosis is associated with the composition and diversity of GM components (Wang et al., 2017; Ling et al., 2021).

Regarding osteoimmunology, GM dysbiosis has been reported to contribute to osteoporosis (Wang et al., 2017; Ibáñez et al., 2019; Ling et al., 2021) but may also affect hyperostosis diseases. Hyperostotic diseases such as OPLL and DISH exhibit a strong association with obesity, type 2 diabetes mellitus, and the complications of metabolic syndrome, which are characterized by systemic low-grade inflammation related to aging (Mader et al., 2021; Kawaguchi, 2022). In addition, OPLL has been reported to be associated with leptin and chronic inflammation (Kawaguchi, 2022). As mentioned, GM dysbiosis affects bone metabolism in association with low levels of chronic inflammation-related diseases and the neurotransmitter leptin, suggesting that it could be a pathological factor not only in osteoporosis but also in hyperostotic diseases such as OPLL. Thus, the term “gut-bone axis” (Zaiss et al., 2019) indicates that GM is associated with bone metabolism *via* nutrient absorption, inflammation immunity, and neurotransmitters, suggesting a strong association between GM dysbiosis and osteoporosis and hyperostotic diseases such as DISH and OPLL. As our understanding of the gut-bone axis’ pathophysiology improves, GM could emerge as a potential treatment option for bone metabolism disorders and low back pain. However, to the best of our knowledge, no epidemiological studies have reported on the association between GM and bone-related SDD such as osteoporotic spinal vertebral fractures or hyperostotic diseases.

### 3.2. Gut-joint axis: OA and facet joint syndrome

**Figure 4:**
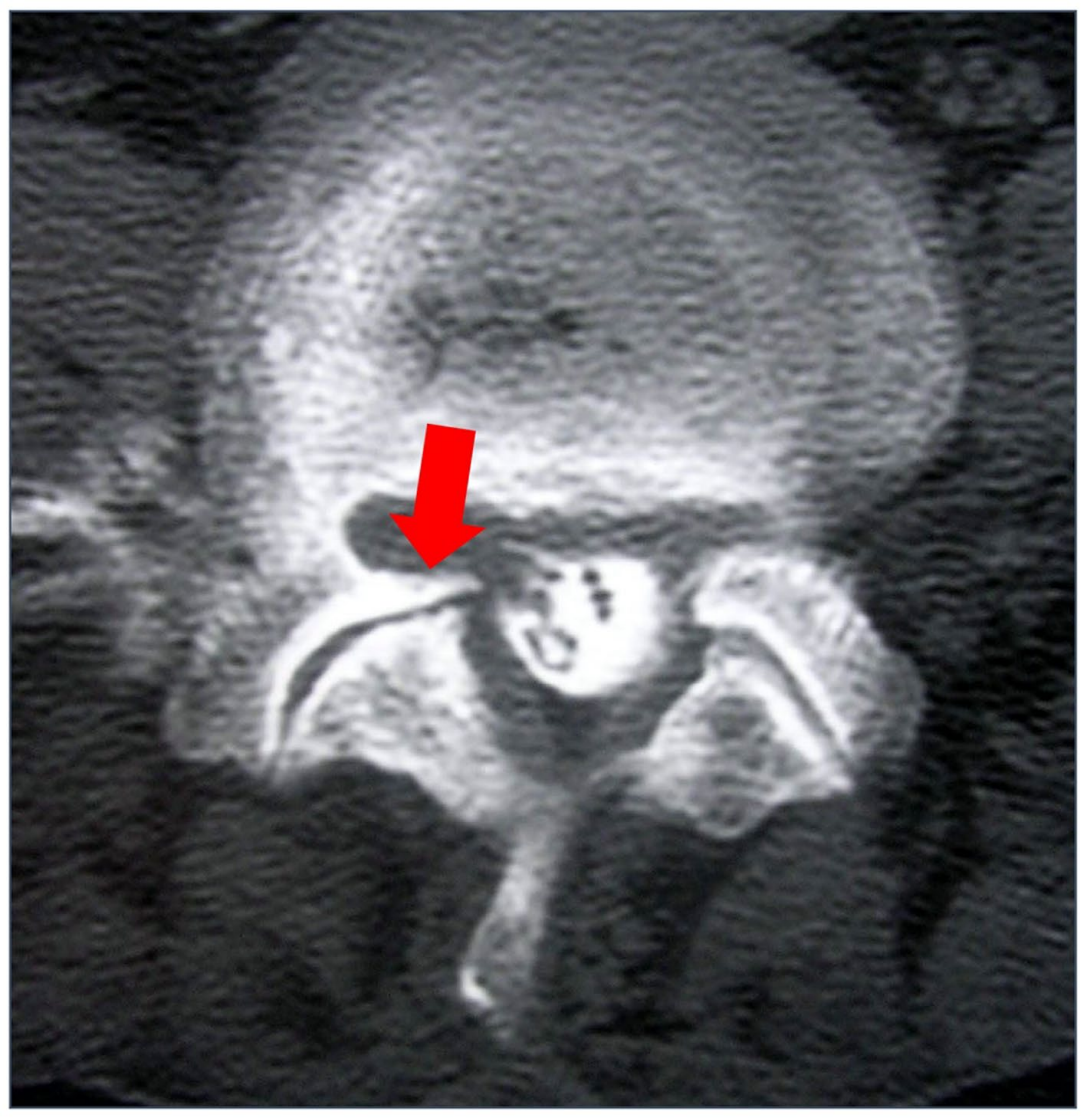
Myelography computed tomography (CT) in lumbar axial view. The red arrow indicates facet degeneration and osteophyte. This is a case from our institution.

OA caused by cartilage degeneration in facet joints has been proposed as a facet joint syndrome among SDD (Du et al., 2022; Figure 4). The “gut-joint axis” has been established based on the possibility of cross-talk between the gut and joint (Berthelot et al., 2019; De Sire et al., 2020; Chisari et al., 2021; Qaiyum et al., 2021).

Unfortunately, no reports have shown an association between facet joint syndrome and GM. However, based on the correlation between GM and lower-extremity OA, we believe that the association between GM and facet joint syndrome could be analogous. Obesity and metabolic syndrome are risk factors for OA not only in the load-bearing joints of the lower extremities but also in non-load-bearing joints (wrist and temporomandibular joints), indicating that systemic chronic low-level inflammation may play a role in its development (Puenpatom and Victor, 2009; Yoshimura et al., 2012). Although mechanical and genetic factors have classically been shown to play a major role in the development of OA, a growing number of reports indicate that low levels of inflammation play an important role in the development and progression of OA (Puenpatom and Victor, 2009; Yoshimura et al., 2012). Low levels of inflammation have been associated with obesity/metabolic syndrome as well as aging, diet, and postmenopausal women (estrogen deficiency), all of which are strongly associated with both OA and GM dysbiosis (Li et al., 2016).

Based on common factors between OA and GM dysbiosis, it seems reasonable that GM dysbiosis is associated with the development of OA. It has been suggested that OA patients show significant GM dysbiosis, revealing a shift in pathogenic microorganisms associated with OA (Huang and Kraus, 2016; Favazzo et al., 2020). In addition, because microbes are present in knee and hip OA, occult or subclinical bacterial infections resulting from bacteremia due to GM dysbiosis may accelerate OA (Dunn et al., 2020). Thus, the concept of the “gut-joint axis” can be applied to facet OA, a subset of SDD. However, further research is required to establish the relationship between the gut-joint axis and facet OA in both basic and clinical settings.

### 3.3. Gut-disk axis: IVDD

The IVD comprises three interrelated structures—namely, the central gelatinous nucleus pulposus, the outer annulus fibrosus, and the upper and lower cartilaginous endplates (Li et al., 2022). Vascular invasion into the IVD, which is generally considered the largest non-vascularized structure in the human body, may be detected in IVDD (Li et al., 2022).

While IVDD is multifactorial, chronic uncontrolled low-grade inflammation is progressively implicated in its etiology (Li et al., 2016). The microenvironment of a healthy IVD is vulnerable to immune surveillance functions (playing the role of sentry) because it has a blood-disk barrier, such as the blood–brain barrier in the central nervous system that keeps the IVD immunodominant and provides protection against systemic infection. Hypoxia and a lack of immune surveillance in the IVD create ideal conditions for the growth of anaerobic bacteria in degenerated disks (Wedderkopp et al., 2009). These bacteria growing on the IVD can recruit more inflammatory cells (e.g., T cells, B cells, dendritic cells, macrophages) *via* the release of inflammatory factors (e.g., IL-6, TNFα; Schirmer et al., 2016). Hence, a damaged IVD can become an ideal site for the growth and proliferation of microbes that evade humoral and cellular immunity, as well as the spread of harmful microbiome metabolites (Li et al., 2022). Therefore, GM dysbiosis has been suggested to possibly cause GM and GM metabolites to migrate into the bloodstream and IVD, causing or exacerbating IVDD (Li et al., 2022).

Rajasekaran et al., 2020 evaluated 24 lumbar IVDs and reported that the microbiome composition of healthy IVD differed from that of degenerative and herniated IVD. Thus, the concept of gut-disk axis (Li et al., 2022) is emerging, which may play an important role in IVDD and low back pain.

### 3.4. Gut-ligament axis: lumbar spinal stenosis

**Figure 5:**
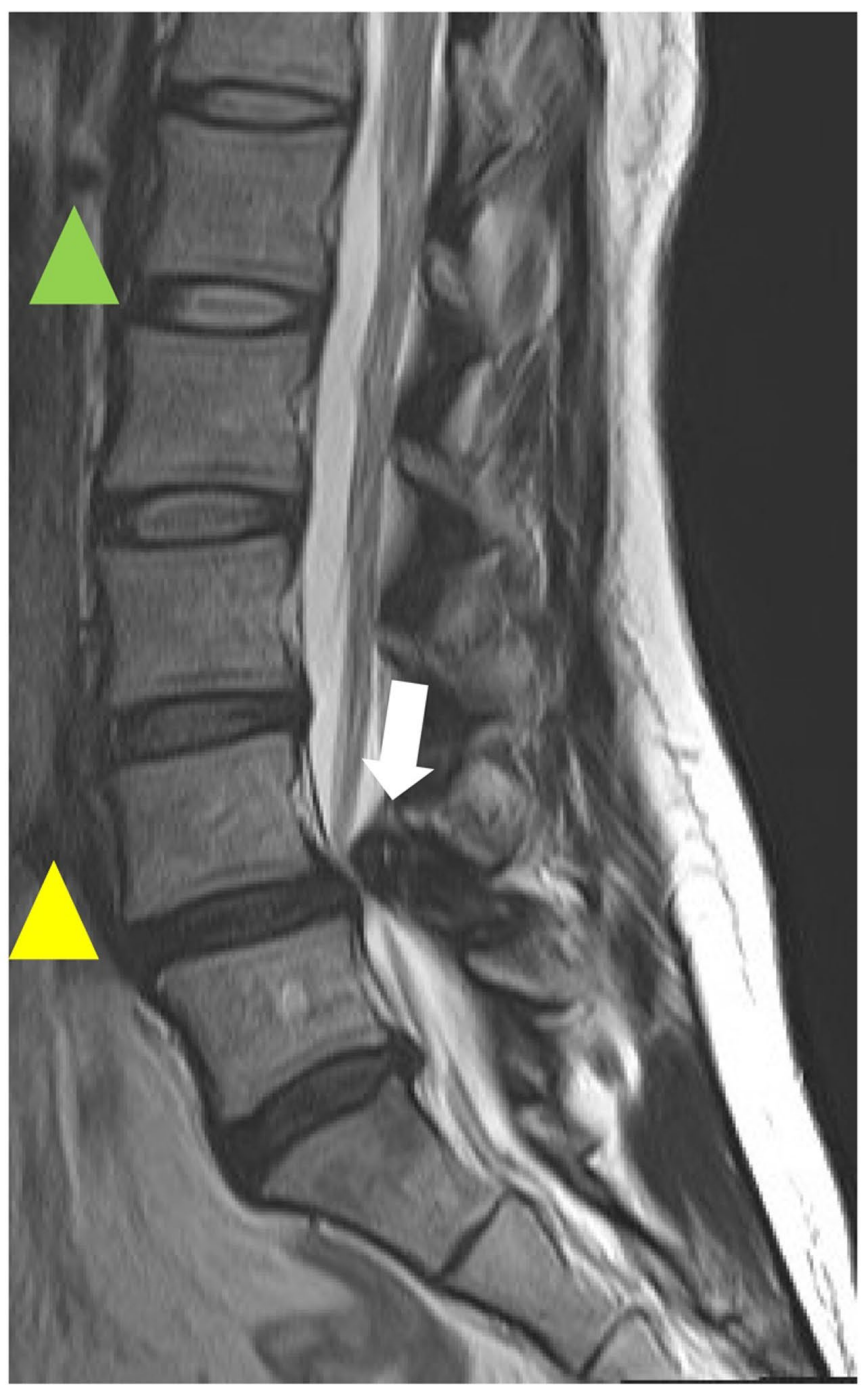
T2-weighted magnetic resonance imaging (MRI) sagittal image of the lumbar spine. The green arrowhead indicates normal intervertebral disk, the yellow arrowhead indicates intervertebral degenerated disk, and the white arrowhead indicates hypertrophic yellow ligament. This is a case from our institution.

In addition to facet joint OA and IVDD, thickening of the yellow lumbar ligament is the leading cause of LSS pathogenesis (Figure 5), and an inflammation-related scar mechanism has been suggested for the thickening of the lumbar ligament (Sairyo et al., 2007). Although an association has been shown between LSS and diabetes, hypertension, and metabolic syndrome, all of which have been closely associated with GM dysbiosis (Fujita, 2021), no reports have shown an association between LSS and GM. However, IVDD and facet OA, which are factors of LSS, have a relationship with GM. Furthermore, the association of LSS with chronic inflammation-related conditions such as diabetes and metabolic syndrome, as well as inflammation observed in ligament thickening among LSS patients, indicates a potential connection between LSS and GM, suggesting the possibility of a gut-ligament axis in LSS.

### 3.5. Gut-muscle axis: spinal sarcopenia

Sarcopenia is defined as a progressive and systemic loss of skeletal muscle mass, strength, and function, according to the European Working Group on Sarcopenia in Older People (Cruz-Jentoft et al., 2019). GM dysbiosis can affect muscle mass and function *via* inflammation, immunity, protein metabolism, SCFA metabolism, and mitochondrial dysfunction, thereby leading to sarcopenia in the spine and ultimately affecting host physiology (Picca et al., 2018). In several studies exploring the correlation between GM and skeletal muscle, significant differences in GM diversity and composition were observed in sarcopenia cases, and a gut-muscle axis has been proposed to indicate cross-talk between the two (Picca et al., 2018; Ticinesi et al., 2019; Kuo et al., 2020; Przewłócka et al., 2020). The number of adult spinal deformities due to spinal sarcopenia (loss of erector spinae) is increasing, and its involvement with GM is an interesting topic of investigation (Kuo et al., 2020).

### 3.6. Impact of GM on SDD-derived pain

The main symptoms of SDD include low back pain and pain or numbness in the upper and lower extremities of nerve origin. Since GM is involved in the production of SCFA, neurotransmitters (including some precursors), and vitamins that regulate inflammation and pain (Benedict et al., 2016; Yan and Charles, 2017; Barton et al., 2018; Lucas et al., 2018), it is plausible that it is strongly involved in SDD-derived pain.

GM produces the neurotransmitters involved in pain modulation and analgesia (including some precursors)–namely, dopamine, serotonin, noradrenaline, glutamate, and GABA (Liu et al., 2020). In addition, the analgesic effects of B vitamins produced by GM are explained by their anti-inflammatory, antinociceptive, and neuroprotective effects (Buesing et al., 2019). The association of abnormal GM composition has also been demonstrated in patients with low back pain (Dekker Nitert et al., 2020), fibromyalgia (Minerbi et al., 2019), chronic pain (Freidin et al., 2021), knee OA (Boer et al., 2019), hand OA (Wei et al., 2021), and peripheral neuropathic pain (Ellis et al., 2022), suggesting that abnormal GM composition may be involved in individual differences in pain sensitivity. However, GM composition abnormalities have not been validated for SDD-related pain.

In addition, analgesics may be used in cases of pain of SDD origin; however, GM strongly interacts with certain drugs, affecting their response and effectiveness (Vich Vila et al., 2020). Especially in cases of long-term opioid use, it has been inferred that adverse changes in GM composition (dysbiosis) can occur and cause predisposition to opioid tolerance (Akbarali and Dewey, 2017). Therefore, understanding the cross-talk between GM and SDD-derived pain may be helpful in understanding the pathogenesis of SDD. In addition, understanding the effect of GM on analgesics used for SDD may explain individual differences in analgesic efficacy and prognosis.

### 3.7. Summary of reports related to GM and spinal musculoskeletal diseases, including SDD

Table 1 presents a summary of the association between GM and spinal musculoskeletal diseases, including SDD (Yang et al., 2016; Wang et al., 2017; Wen et al., 2017; Shen et al., 2019; Rajasekaran et al., 2020; Kang et al., 2021; Ling et al., 2021).

**Table 1:** Gut microbiota and spinal musculoskeletal diseases.

Area	Spinal musculoskeletal diseases	Characteristics of gut microbiota	Evidence
Bone	Osteoporosis	Positive correlation: *Actinobacillus*, *Blautia*, *Oscillospira*, *Bacteroides*, *Phascolarctobacterium*	Ling et al. (2021)
	Osteoporosis	Higher proportion of *Blautia* and *Parabacteroides* and lower proportion of *Ruminococcaceae* UCG-002	Wang et al. (2017)
Joint	Facet joint osteoarthritis	No report	
Disk	Intervertebral disk disease	More frequently or specifically: *Pseudomonas veronii*, *Pseudomonas stutzeri*, *Streptococcus anginosus*, *Prevotella pallens*, *Avibacterium gallinarum*, *Enterobacter cowanii* Many known human pathogens such as *Prevotella tannerae*, *Halomonas nitritophilus*, and *Streptococcus alactolyticus*	Rajasekaran et al. (2020)
Muscles	Sarcopenia	Reduced microbial diversity, with an increased level of *Lactobacillus* and decreased levels of *Lachnospira*, *Fusicantenibacter*, *Roseburia*, *Eubacterium*, and *Lachnoclostridium*	Kang et al. (2021)
Others	Adolescent idiopathic scoliosis	The *Prevotella* genus showed considerable increase in proportion.	Shen et al. (2019)
	Spondyloarthritis	A significantly increased abundance of *Prevotella melaninogenica*, *Prevotella copri*, and *Prevotella* sp. C561 and decreased representation of *Bacteroides* spp.	Wen et al. (2017)
	Ankylosing Spondylitis	*Porphyromonas gingivalis*, *Klebsiella pneumoniae*, *Klebsiella aerogenes*, and *Bacteroides vulgatus* are associated with pathogenesis	Yang et al. (2016)

As a combination of specific microbiota abundance and SDD, Blautia and osteoporosis (Wang et al., 2017; Ling et al., 2021), Prevotella and Intervertebral disk disease (Rajasekaran et al., 2020), and Adolescent idiopathic scoliosis (Shen et al., 2019), Spondyloarthritis (Wen et al., 2017).

SDD usually first results from degeneration of IVD and facet joints, i.e., IVDD or facet OA develops. As a result, the stabilizing function between intervertebral bodies is impaired, which leads to the formation of bony spurs around intervertebral bodies and facet joints, and the thickening of the yellow ligament, resulting in LSS, including lumbar spondylolisthesis. In addition, spinal sarcopenia and OVF can accelerate the pathology. Moreover, SDD-related pain derived from IVDD, facet OA, LSS, and OVF impairs activity, further aggravating GM dysbiosis, sarcopenia, and osteoporosis. Thus, osteoporosis, IVDD, facet OA, LSS, and spinal sarcopenia coexist, adversely affecting not only SDD but also GM dysbiosis, leading to a negative spiral. With the advent of an aging society, understanding the pathophysiology, prevention, and treatment of SDD will become increasingly important. We have discussed that GM affects all pathologies of SDD and SDD-derived pain. Investigating the profile of GM in the progression of SDD could help identify patients with rapidly progressive SDD and improve our understanding of the pathogenesis of SDD.

## 4. Gut microbiota modulation as treatment for SDD

Given the worldwide prevalence of SDD, there is a critical need for effective disease-modifying treatment strategies to alleviate symptoms and slow SDD progression.

GM communicates with the distant spinal structures *via* various axes, such as the gut-bone axis, gut-joint axis, gut-disk axis, gut-ligament axis, and gut-muscle axis. Bacteremia and chronic low-level inflammation caused by GM dysbiosis adversely affect spinal structures (bones, cartilage, disks, ligaments, and muscle) and contribute to the onset and progression of SDD, including osteoporosis, IVDD, and OA. Conversely, restoring GM dysbiosis through therapeutic intervention may restore physiological regulation through various axes and prevent the onset and progression of SDD (Figure 2).

GM has attracted attention as a promising target for therapeutic strategies because it can be modified by lifestyle modifications such as dietary interventions, sleep and exercise, fecal transplants, and future microbiome-targeted therapies (Kolasinski et al., 2020).

### 4.1. Lifestyle interventions (diet, sleep, exercise) for GM dysbiosis may improve SDD

Given the association between GM dysbiosis and SDD and chronic inflammation, GM dysbiosis may be involved in the development and progression of SDD. As discussed in Chapter 2 (Li et al., 2022), a healthy lifestyle (including factors such as diet, sleep, and exercise) improves GM composition (increases “good” bacteria) and may improve GM dysbiosis. An improvement in GM dysbiosis may also alleviate inflammation, inhibit the degeneration of spinal structures, and relieve pain via SCFA or neurotransmitters. The interaction between lifestyle (diet, sleep, exercise) and GM has contributed to our understanding of the role of lifestyle in the prevention and treatment of chronic inflammation-related diseases, including SDD, in which GM dysbiosis plays a significant role. Notably, dietary interventions involving prebiotics and probiotics have shown promising effects in managing osteoporosis and OA via GM modulation (Vitetta et al., 2013; Zhang et al., 2022a).

Prebiotics are non-digestible food components such as dietary fiber and oligosaccharides. Prebiotics stimulate the growth and/or activity of beneficial bacteria in the digestive tract in ways that are beneficial to health, induce SCFA synthesis, affect cell growth and differentiation, hormone production, and inflammation regulation, and have a beneficial effect on the host (Vitetta et al., 2013; Zhang et al., 2022a). Probiotics consist of live microorganisms, typically lactic acid bacteria that modulate protease-activated receptor expression in epithelial cells, gastrointestinal smooth muscle cells, and capsaicin-sensitive neurons to regulate gastrointestinal mucosal barrier function and inflammation (Vitetta et al., 2013; Zhang et al., 2022a). As a result, they play an important role in the homeostasis of healthy GM by promoting epithelial barrier function and reducing dysbiosis, stimulating the production of antimicrobial substances and immunoglobulins, and inhibiting the production of bacterial toxins, thereby promoting host immune responses and anti-inflammatory pathways (Vitetta et al., 2013; Zhang et al., 2022a).

Both prebiotics and probiotics have been reported to have anti-inflammatory effects, promote calcium and vitamin D absorption, reduce osteoclast differentiation, and protect the bone and cartilage. They have also shown beneficial effects on osteoporosis and OA (Table 2; Abrams et al., 2005; Jones et al., 2013; Vitetta et al., 2013; Lei et al., 2017; Takimoto et al., 2018; Lyu et al., 2020; Paul et al., 2021; Zhang et al., 2022a). Nevertheless, no reports on their effects on IVD or LSS have yet been published.

**Table 2:** Prebiotics and probiotics for musculoskeletal diseases in human.

Prebiotics/Probiotics	Area	Musculoskeletal diseases	Comments	Evidence
Prebiotics				
	Bone			
		Osteoporosis	Prebiotic short- and long-chain inulin type fructans significantly increases calcium absorption and enhances bone mineralization	Abrams et al. (2005)
Probiotics				
	Bone	Osteoporosis	*Bacillus subtilis* C-3102 (C-3102), total hip BMD improved	Takimoto et al. (2018)
		Osteoporosis	*Lactobacillusreueri* NCIMB 30242 increases mean circulating 25-hydroxyvitamin D	Jones et al. (2013)
		Osteoporosis	*Lactobacillus reueri* reuteri NCIMB 30242 increases the mean circulating 25-hydroxyvitamin D level	Jones et al. (2013)
	Joint	Osteoarthritis	*Lactobacillus casei* strain Shirota: a positive effect of improvement in knee OA	Lei et al. (2017)
		Rheumatoid arthritis	*Lactobacillus casei* 01 improved the inflammation status	Paul et al. (2021)

Regarding exercise interventions, focusing on the “gut-muscle” axis and spinal sarcopenia, GM has been shown to improve skeletal muscle mass and function, while exercise affects GM composition (Locantore et al., 2020). In addition, the gut-spine axis can be considered as a result of treatment for spinal structures degeneration (bones, cartilage, disks, ligaments, and muscles), in which improvement in exercise level leads to improvement in GM composition. This positive spiral relationship between exercise, GM, and spinal structures could be useful in the prevention and treatment of SDD.

Sleep disturbances promote GM dysbiosis (decreased intestinal bacterial diversity and anti-inflammatory intestinal bacteria), which induces systemic chronic inflammation, which has been reported to be a risk factor for chronic inflammation-related diseases (diabetes and obesity). Chronic inflammation has been associated with the onset and progression of SDD; therefore, the association between sleep disturbances and SDD cannot be ruled out. Conversely, healthy sleep can be expected to contribute to SDD improvement. However, no studies have reported on the effects of sleep on the development or progression of SDD or its preventive effects.

GM also plays an important role in drug metabolism, which may influence drug efficacy (Vich Vila et al., 2020). Hence, GM modulation through lifestyle modification may enhance the efficacy of analgesics and contribute to drug reduction. Drug reduction is a critical issue in SDD patients, mostly older individuals because polypharmacy is also a significant problem. Regulation of GM through lifestyle improvement may also be effective in this regard.

Maintaining a healthy lifestyle is well understood to be based on good nutrition, regular exercise, and adequate sleep. However, only few studies have investigated the correlation between a healthy lifestyle and SDD in the field of spine surgery.

The GM-mediated therapeutic effect of lifestyle interventions is both a new perspective on SDD treatment and effective for SDD. Large-scale clinical trials on lifestyle interventions are required.

### 4.2. Fecal microbiome transplant

FMT is a method of improving certain medical conditions such as GM dysbiosis-related diseases of three types of inflammation: acute inflammation (e.g., *Clostridioides difficile* infection), chronic inflammation (e.g., chronic Crohn’s disease and ulcerative colitis), and chronic low-grade inflammation by delivering specially prepared stool material from a healthy donor to the patient (i.e., recipient) and restoring the balance of the intestinal bacterial community (Wang et al., 2022; Zhang et al., 2022b).

In contrast to applications targeting single microorganisms, such as probiotics and prebiotics, FMT maintains the integrity of GM and metabolites during the entire process, thus preserving the original function of GM to the maximum extent possible, significantly improving GM-related diseases and restoring gut microenvironmental homeostasis more rapidly and efficiently (Zhang et al., 2022b). While physiological disturbances due to disease can alter the composition and abundance of GM, GM dysbiosis can, on the other hand, induce or exacerbate disease, and FMT can be expected to prevent or alleviate disease conditions.

The potential regulatory mechanisms involving the FMT are to reestablish a normal intestinal environment and to correct disturbances in the intestinal microbiota. It is believed to potentially restore the intestinal mucosal barrier, improve intestinal permeability, restore imbalances in intestinal metabolites (SCFA, indole derivatives, vitamins, cholic acid, polyamines), and regulate immune responses (Zhang et al., 2022b).

FMT has been performed and validated for GM dysbiosis-related diseases in (1) Gastrointestinal diseases: Bacterial intestinal infection (*Clostridioides difficile* infection), inflammatory bowel disease (Crohn’s disease and ulcerative colitis), and irritable bowel syndrome, (2) Liver Diseases: Severe alcoholic hepatitis, primary sclerosing cholangitis, and liver cirrhosis, (3) Brain diseases: Autism spectrum disorder, Parkinson’s disease, multiple sclerosis, Alzheimer’s disease, and epilepsy, (4) Metabolic diseases: Diabetes, obesity, metabolic syndrome, and gout, (5) Cancer: Melanoma, and gastroesophageal cancer, (6) Skin diseases: alopecia and atopic dermatitis (Wang et al., 2022; Zhang et al., 2022b).

Spinal musculoskeletal disorders in which FMT has been reported to be effective are inflammatory, immune, or metabolic diseases: osteoporosis (Zhang et al., 2022b), psoriatic arthritis (Selvanderan et al., 2019), and axial arthritis (Mahajan et al., 2020). For other SDD (OA, IVDD, LSS, spinal sarcopenia) for which the effects of FMT have not yet been reported, GM dysbiosis may increase the risk of development and progression, and further normalization of GM composition and function may contribute to prevention, treatment, and symptom improvement.

## 5. Conclusion and perspective

The close relationship between GM and spinal structures (bones, cartilage, disks, ligaments, and muscles) due to common factors such as immunity, metabolism, and inflammation has been described by the terms gut-bone axis, gut-joint axis, gut-disk axis, gut-ligament axis, and gut-muscle axis.

Bacteremia and chronic low-level inflammation caused by GM dysbiosis have been found to adversely affect spinal structures and contribute to the development and progression of SDD, including osteoporosis, LSS, IVDD, spinal sarcopenia, and SDD-derived pain. Since GM-derived neurotransmitters may regulate the excitability of neurons in the peripheral nervous system and nociceptors involved in the onset of SDD-derived pain, GM may modulate the pathogenesis and therapeutic effects of SDD. This close association between GM and SDD led us to propose the gut-spine axis.

Scientific progress is often driven by the clear demarcation of research areas; however, to understand the complex and multifaceted nature of SDD, it is necessary to integrate the findings of various disciplines, such as spinal anatomy, immunology, microbiology, aging, and more. The perspective provided by GM research is a good example of such interdisciplinary integration and may not only provide a new framework for understanding these biological systems but may also offer many valuable insights into the development of effective therapies for the treatment of SDD (new disease-modifying therapies that intervene in GM). New biomarkers associated with inflammation and gut dysbiosis may predict the development of SDD and monitor the effectiveness of therapeutic interventions. Chronic inflammation and adverse immune effects due to GM dysbiosis and bacteremia (joint and IVD are not always sterile) may also contribute to postoperative infection of SDD.

However, the literature on the relationship between GM and SDD is scarce due to various issues such as cost, time, and declining participation, suggesting that there is an existing knowledge gap between the two. While a healthy lifestyle (diet, sleep, exercise) and FMT may improve GM dysbiosis and consequently contribute to SDD improvement, few reports exist on this issue, and large-scale studies are therefore needed.

Preventive medicine is important because increasing SDD is an urgent problem in an aging society. Preventive medicine is divided into three categories: primary prevention (health promotion, anti-aging medicine, prevention of diseases), secondary prevention (early detection, early treatment, prevention of aggravation), and tertiary prevention (rehabilitation, prevention of recurrence). GM is strongly related to all these SDD prevention aspects. The Nobel Prize-winning discovery of the bacterium *Helicobacter pylori* as the primary cause of gastritis and peptic gastritis was a game changer in diagnosis and treatment, radically shifting the management of peptic ulcers from surgical treatment to antibiotics and acid secretion inhibitors (Rajasekaran et al., 2020).

A better understanding of GM could be the catalyst for a new game changer in the prevention, diagnosis, and treatment of SDD, with GM as the axis. The established association between GM and spinal structures (gut-spine axis) supports the feasibility of a new approach to the prevention, diagnosis, and treatment of SDD.

## Author contributions

TM: Data curation, Visualization, Writing – original draft, Writing – review & editing. TaK: Investigation, Methodology, Writing – original draft. ToK: Project administration, Resources, Writing – review & editing. ME: Supervision, Writing – review & editing. MT: Formal analysis, Writing – review & editing. TY: Formal analysis, Data curation, Visualization, Writing – review & editing. HH: Resources, Visualization, Writing – review & editing. MM: Supervision, Writing – review & editing. SY: Resources, Writing – review & editing.

## Abbreviations

DISH: diffuse idiopathic skeletal hyperostosis
GM: gut microbiota
IVD: intervertebral disk
IVDD: intervertebral disk degeneration
LPS: lipopolysaccharide
LSS: lumbar spinal canal stenosis
NGS: next-generation high-throughput microbial DNA sequencing
OA: osteoarthritis
OPLL: ossification of posterior longitudinal ligament
OVF: osteoporotic vertebral fractures
SASP: senescence-associated secretory phenotype
SCFA: short-chain fatty acids
SDD: spinal degenerative diseases
